# Supplementary material for: Individualized precision targeting of dorsal attention and default mode networks with rTMS in traumatic brain injury-associated depression
Source: Sci Rep. 2023 Mar 11;13:4052. doi: 10.1038/s41598-022-21905-x (PMC10008633; doi:10.1038/s41598-022-21905-x)
Supplement: Supplementary file 1 — Supplementary Information. [file 41598_2022_21905_MOESM1_ESM.docx]

**Individualized precision targeting of dorsal attention and default mode networks with rTMS in traumatic brain injury-associated depression**

**Supplementary material**

Contents

[Supplementary methods 2](#_Toc31863534)

[1. Standard protocol approvals and participants 2](#_Toc31863535)

[2 MRI acquisition and pre-processing 2](#_Toc31863536)

[2.1 Acquisition 2](#_Toc31863537)

[2.2 BOLD Pre-processing 3](#_Toc31863538)

[2.3 BOLD Post-processing 3](#_Toc31863539)

[3 rTMS target selection and comparison 3](#_Toc31863540)

[3.1 Confirmation of candidate network targets 3](#_Toc31863541)

[3.2 Individualized RSN-based target selection 3](#_Toc31863542)

[Figure S1: Algorithm for individualized target selection. 4](#_Toc31863543)

[3.3 Alternative target selection 4](#_Toc31863544)

[3.4 Functional connectivity comparison of targeting methods 5](#_Toc31863545)

[4 Treatment-induced changes 5](#_Toc31863546)

[4.1 rTMS treatment parameters 5](#_Toc31863547)

[4.2 Treatment-induced connectivity changes 5](#_Toc31863548)

[Supplementary results 7](#_Toc31863549)

[Table S1: Individual ANOVA results for connectivity with DAN, DMN, and the sgACC seed map. 7](#_Toc31863550)

[Table S2: Variance in coordinates identified by each individualized targeting algorithm. 7](#_Toc31863551)

[Figure S2: Stimulation site connectivity and antidepressant response. 8](#_Toc31863552)

[Figure S3: Change in stimulation site connectivity with Yeo network maps and with whole brain. 9](#_Toc31863553)

[Supplementary References 10](#_Toc31863554)

# **Supplementary methods**

1. Standard protocol approvals and participants
The study was approved by the Human Research Protection Office at Washington University School of Medicine in St. Louis. All individuals gave informed written consent. The study was registered with ClinicalTrials.gov (NCT02980484). Methods and hypotheses were pre-registered with the Open Science Foundation (osf.io/vjddq)[1]. The study was reviewed regularly by the investigators for safety, including weekly group meetings.

15 subjects (11 males, ages 19-64) with TBI-D were recruited from the Washington University TBI clinics as part of a pilot randomized-controlled trial of RSNM-targeted rTMS. This included patients with clinically significant depression as quantified by a score of at least 10 on the Montgomery-Asberg Depression Rating Scale (MADRS) and a history of at least one concussive or moderate TBI associated with low risk of seizure disorder. Patients with penetrating head injury were excluded due to seizure risk. The clinical results of the trial and detailed patient characteristics are reported elsewhere[2]. This analysis was limited to 13 subjects (10 males) who completed both the pre-treatment and post-treatment scan sessions. 11 of these 13 subjects had concussive TBI, while 2 of 13 had moderate TBI. Six of the 13 subjects had comorbid post-traumatic stress disorder. All subjects had failed multiple antidepressant medications, with a mean of 3.7 ± 1.9 failed medications. There were no stipulations imposed regarding whether the depressive symptoms occurred or worsened subsequent to TBI, and we made no attempts to determine the causal relationship between TBI and depressive symptoms.

10 healthy control subjects (3 males, ages 22-35) with no history of neuropsychiatric disease received rsfMRI scans as part of the Human Connectome Project (HCP)[3]. These subjects were chosen randomly from the HCP database. The mean age was lower in the healthy control group because older adults were not included in the original HCP database.

## 2 MRI acquisition and pre-processing

#### 2.1 Acquisition

For TBI-D subjects, functional and anatomical images were acquired with a 3T Siemens Magnetom Prisma magnetic resonance scanner (Siemens, Erlangen, Germany) located at Washington University in St. Louis. The 16.5 minute scan duration was chosen because it approximates the point at which reliability of resting-state functional connectivity estimates approaches an asymptote[4, 5]. BOLD scan parameters included 416 frames per run, 48 axial slices using 4-band acquisition, 3 mm isotropic voxel resolution, repetition time (TR) 800 ms, echo time (TE) 26.6 ms, flip angle 61 degrees, and imaging matrix 72 x 72. A T1 MPRAGE structural sequence was acquired with 176 slices, 0.9375 x 0.9375 x 1 mm voxel resolution, TR 2400 ms, TE 3.19 ms, flip angle 8 degrees, and imaging matrix 256 x 256.

For HCP subjects, images were acquired using the 3T Connectome Skyra, a modified Siemens Magnetom Skyra at Washington University with a customized high-gradient insert and body transmitter coil. Acquisition included 58 minutes of resting-state blood oxygen-level dependent (BOLD) scans in four runs (1200 frames per run, 72 axial slices using 8-band acquisition, 2 mm isotropic voxel resolution, repetition time (TR) 720 ms, echo time (TE) 33.1 ms, flip angle 52 degrees, imaging matrix 104 x 90) in addition to a T1 MPRAGE structural sequence (192 slices, 0.7 mm isotropic voxel resolution, TR 2400 ms, TE 2.14 ms, flip angle 8 degrees, field of view 224 x 224 mm).

#### 2.2 BOLD Pre-processing

Spatial alignment and common Talairach atlas registration were performed using the 4dfp suite of tools developed at Washington University. All subsequent analyses were performed in Talairach atlas space except as otherwise specified. Anatomical segmentation and surface reconstruction was conducted on each subject’s anatomical T1-weighted scan using FreeSurfer version 5.3.0, HCP release[6]. HCP data were resampled from 2mm to 3mm isotropic voxel resolution. Motion censoring, nuisance regression, global signal regression, temporal filtering, spatial smoothing, and motion epoch interpolation were performed using in-house scripts described in Power et al., 2014. Framewise displacement of 0.5 mm was used for motion censoring because the noise floor of FD values for some subjects was higher than what is typically reported, as has been previously described for data with fast TR[7]. Detailed description of head motion characteristics have been presented previously[8].

#### 2.3 BOLD Post-processing

BOLD time courses were used to construct individual-level RSN maps via a multilayer perceptron (MLP)-based machine learning classifier as described in Hacker et al., 2013[9]. Briefly, the MLP was initially trained on a reference set of correlation maps derived from seed locations identified by task meta-analyses. For new subjects, the trained classifier uses whole-brain correlation analyses to determine the likelihood of each voxel’s membership in one of seven RSNs, including dorsal attention network (DAN), ventral attention network (VAN), frontoparietal control network (FPCN), default mode network (DMN), sensorimotor network (MOT), language network (LAN), and visual cortex (VIS). Of note, VAN in this parcellation corresponds with cingulo-opercular and salience networks in some other cortical parcellations[9]. Surface projections were visualized on the Conte69 atlas brain using Connectome Workbench v1.0[10].

Using the voxel-wise estimates for each of the seven individualized resting-state network maps, each voxel was assigned to the network with which it exhibited the maximum likelihood of membership. These values were used to construct a “winner-take-all map” of cortical parcels. Each of the seven networks were extracted from this map in order to define the parcellated bounds of each network.

## 3 rTMS target selection and comparison

#### 3.1 Confirmation of candidate network targets

Group connectome data from the HCP 800-subject release[3] were used to construct normative maps of resting-state functional connectivity (figure 1a). A seed was chosen at sgACC, MNI coordinates (6,16,-10), due to the known association between rTMS efficacy and normative connectivity profile of the stimulation site with this coordinate[11]. In comparison with other resting-state networks, DAN and DMN maps were expected to jointly demonstrate the closest approximation of group-based sgACC seed maps.

#### 3.2 Individualized RSN-based target selection

Based on the individualized RSN maps, an additional map was computed to reflect the difference between likelihoods of DAN and DMN membership for each voxel (figure S1a). In order to incorporate only superficial regions that are readily accessible via rTMS, the image was masked to include only voxels within 6 mm of the outer surface. As an approximation of DLPFC, a second mask was applied to include only voxels within 20 mm of previously-reported coordinates[12] for Brodmann areas 9 and 46 (figure S1b); while previous retrospective work has utilized a 25 mm radius[12], this was less practical for a prospective study due to inclusion of excessively anterolateral regions (which are more likely to produce uncomfortable facial muscle contraction) and excessively posterior regions (which are associated with increased seizure risk due to possible inadvertent stimulation of motor cortex). Positive clusters in the resulting image were identified using FSL’s cluster algorithm (FMRIB Software Library, Oxford, UK)[13, 14] with an image threshold of 75% of the maximum z-score. The centers of gravity of the peak clusters in each hemisphere were chosen as optimal left- and right-sided rTMS stimulation sites (figure S1c). These coordinates were transformed from Talairach to native space using the 4dfp tool suite.

**Figure S1: Algorithm for individualized target selection.** (a) Example of an individualized map of the difference between DAN and DMN membership likelihood. Red areas indicate high DAN and low DMN membership likelihood, while blue areas indicate low DAN and high DMN membership likelihood (b) Mask of dorsolateral prefrontal regions (red) and regions within 6 mm of the brain surface (white). (c) Final masked DAN minus DMN membership likelihood map; the center of gravity in this map (arrow) was used as the individualized treatment target.


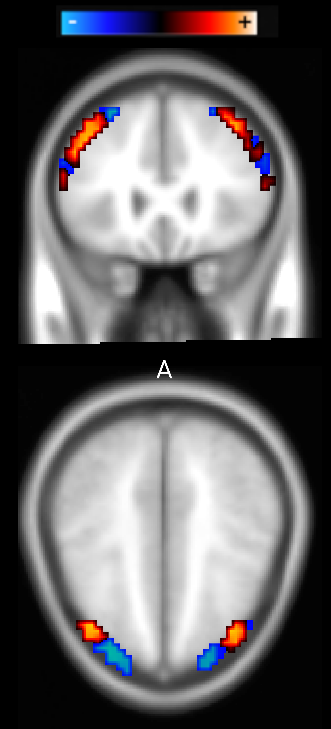

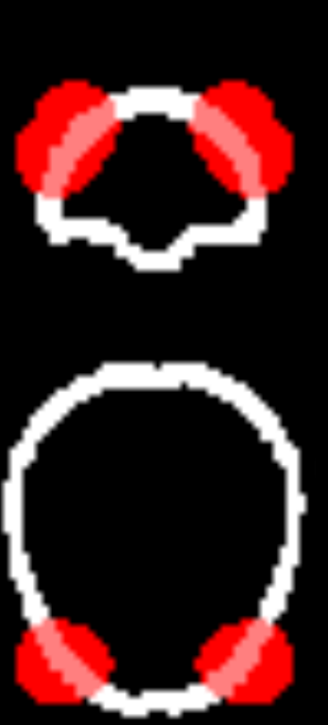

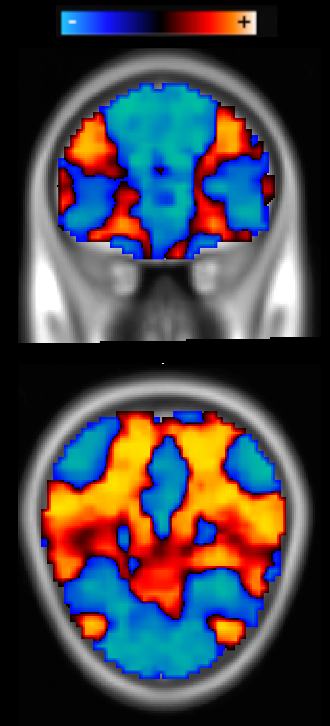


**(a) (b) (c)**

#### 3.3 Alternative target selection

Alternative target coordinates were generated using structural MRI-based targeting and individualized anti-sgACC targeting.

Structural MRI-based targeting coordinates were based on the dorsolateral prefrontal target that has been used at the world’s current largest neuronavigated rTMS clinic[15]. While other anatomical targets have been used in the past, the recent consensus is converging towards the use of coordinates where group-mean connectivity analysis shows maximum anticorrelation with sgACC[12]. The left- and right-sided targets were chosen at the MNI coordinates (±38, 44, 26).

Individualized anti-sgACC targeting was based on each individual subject’s anti-correlation with group average sgACC coordinates following the methods described by Fox et al., 2013[16]. Spherical seeds with 10 mm diameter were selected at MNI coordinates (±6, 16, -10) for left and right sgACC[16]. Whole-brain correlation maps for each were masked to include only cortical regions within 20 mm of previously-reported coordinates for Brodmann areas 9 and 46[16]. The peak negative cluster was identified using the FSL cluster algorithm[17]. The center of this cluster was considered to be the “anti-sgACC” rsfMRI-based target. When this method generated multiple targets, the optimal target was conservatively chosen as the coordinate with largest DAN-DMN correlation difference determined using the method described in section 2.4.3. This choice was considered conservative because it is least likely to be different from the DAN-DMN target.

#### 3.4 Functional connectivity comparison of targeting methods

The rTMS stimulation volume for each of the three targets was estimated as a sphere with 15 mm diameter centered at the target coordinates; while there is no consensus regarding the optimal field modeling technique for functional connectivity analyses[18], prior studies have utilized estimates in the range of 12 – 20 mm[12, 16, 19]. This stimulation volume was used as a seed to calculate resting-state functional connectivity with DAN, DMN, and the normative sgACC seed map. DAN was defined as Yeo parcels 5/6 and DMN was defined as Yeo parcels 16/17 based on the 17-network parcellation in Yeo et al., 2011[20]. Parcel 12, which contains some DAN components, was excluded because of substantial overlap with the prefrontal treatment site, which could bias the analysis. Parcel 15, which includes regions traditionally associated with DMN, was excluded because recent parcellations have defined it as a separate network[21]. The sgACC seed map was treated as a weighted seed, as per the methods in Fox *et al*, 2013[16]. These group-mean definitions, rather than the Hacker et al. definitions[9], were used for the purpose of conservative prediction of network stimulation profiles, as target correlation with individualized DAN and DMN parcels would naturally be higher for target seeds defined based on the same individualized parcels.

To confirm that this effect was not driven by autocorrelation between the RSNM-based DAN/DMN parcels and the Yeo DAN/DMN parcels, connectivity was also calculated with the normative sgACC seed map. If the effect was driven by this autocorrelation, then presumably the sgACC seed map would be most correlated with the anti-sgACC targets.

## 4 Treatment-induced changes

#### 4.1 rTMS treatment parameters

Treatment was delivered using a Magstim Rapid-2 stimulator and was targeted using a Brainsight neuronavigation device. Using the Brainsight software, each subject’s target coordinates were plotted on a surface reconstruction of that subject’s brain. Four landmarks on the patient’s face (left tragus, right tragus, nasion, and tip of nose) were cross-registered to the MRI scan. The TMS coil was placed at an angle and position to minimize the coil-to-cortex distance as well as the distance between the actual TMS target and the optimal TMS target. The coil position was manually monitored throughout the course of each treatment to confirm that the actual TMS target remained within 5 mm of the optimal target coordinate.

#### 4.2 Treatment-induced connectivity changes

The general approach to calculation of treatment-induced connectivity changes is described in the main text. Detailed parameters are described here.

For voxel-wise analyses, multiple comparisons correction was conducted using the conservative approach to cluster-based inference described by Slotnick *et al*, 2017[22]. The minimum cluster-extent threshold generated by this algorithm (351 mm^3^ with detection threshold of p<0.001) was used to detect clusters with corrected p<0.05. While recent work by Eklund *et al.* has questioned the validity of liberal approaches to cluster-based multiple comparisons correction, this work did find that more conservative approaches yielded acceptable family-wise error rates[23]. Our correction approach was substantially more conservative than these acceptable methods due to the use of a relatively large cluster extent threshold while maintaining a conservative detection threshold.

A similar approach was used for multiple comparisons correction in the voxel-wise prediction of antidepressant response. Because sample size was reduced by limiting the analysis to the active treatment group, the cluster detection threshold was defined more liberally (p<0.01), leading to a recommended extent threshold of 729 mm^3^ using the Slotnick *et al* algorithm[22]. This minimum threshold was doubled in order to maintain conservative inference. To provide additional confirmation of this approach, statistical hypothesis testing was also conducted using permutation testing with 10,000 iterations. The full analysis was repeated after randomly permuting the antidepressant response scores. Significance was assessed based on the number of significant voxels in these permuted cluster maps in comparison with the number of significant voxels in the real cluster maps.

# **Supplementary results**

#### ***Table S1:*** *Two-way ANOVA results for connectivity with DAN, DMN, and the sgACC seed map.*

There was a significant effect of targeting method on target connectivity with DAN, DMN, and sgACC seed map in both groups. The targeting method and side (left vs. right) were both treated as repeated measures.
Comparison between individual targeting methods is reported as the difference in the main effect of each pair of targeting methods (after Tukey’s correction for multiple comparisons). In comparison to the anti-group mean sgACC method, RSNM targets showed significantly stronger correlation with all three target networks in the TBI-D cohort, and with two of the three target networks in the HCP cohort. In comparison to the structural method, RSNM targets showed significantly stronger correlation with two of the three target networks in the TBI-D cohort. The structural targets and anti-group mean sgACC targets showed significant differences in connectivity to DMN in the TBI-D cohort, but no significant differences in the other 5 analyses.

| **Group** | **Connectivity with:** | **Effect of targeting method** | **Effect of side (Left vs. Right)** | ***p*** (Main effect of RSNM vs. Anti-group mean sgACC) | ***p*** (Main effect of RSNM vs. Structural) | ***p*** (Main effect of Structural  vs. Anti-group mean sgACC) |
| --- | --- | --- | --- | --- | --- | --- |
| **TBI-D** (n=26 targets, n=13 subjects) | **DAN** | F(2,24) = 7.5 p = 0.003 | F(1,12) = 0.003 p = 0.96 | 0.004 | 0.02 | 0.82 |
|  | **DMN** | F(2,24) = 5.8 p = 0.009 | F(1,12) = 0.03 p = 0.86 | 0.01 | 0.91 | 0.03 |
|  | **sgACC seed map** | F(2,24) = 9.5 p = 0.0009 | F(1,12) = 0.18 p = 0.68 | 0.002 | 0.003 | 0.98 |
| **HCP** (n=20 targets, n=10 subjects) | **DAN** | F(2,18) = 3.9 p = 0.04 | F(1,9) = 3.9 p = 0.08 | 0.03 | 0.27 | 0.49 |
|  | **DMN** | F(2,18) = 6.4 p = 0.008 | F(1,9) = 1.8  p = 0.22 | 0.007 | 0.10 | 0.54 |
|  | **sgACC seed map** | F(2,18) = 3.4 p = 0.06 | F(1,9) = 4.3  p = 0.07 | 0.07 | 0.13 | 0.94 |

#### ***Table S2:*** *Variance in coordinates identified by each individualized targeting algorithm.*

On average, the RSNM targets and anti-sgACC targets identified similar coordinates. However, RSNM targets showed significantly less variance between subjects, especially in the TBI depression group. The two cohorts were not directly compared with one another due to differences in acquisition methods.

|  | **RSNM** | | **Anti-group mean sgACC** | | **F-test for difference  in variance** |
| --- | --- | --- | --- | --- | --- |
|  | *Mean coordinates* | *Distance from mean* | *Mean coordinates* | *Distance from mean* |  |
| **TBID** | Left: (-44, 37, 24) Right: (43, 38, 27) | 5.1 ± 8.4 mm | Left: (-44, 39, 23) Right: (42, 41, 24) | 13.9 ±  26.9 mm | F = 0.4 p = 0.01 |
| **HCP** | Left: (-41, 40, 26) Right: (40, 41, 27) | 5.2 ±  5.1 mm | Left: (-37, 38, 22) Right: (41, 36, 22) | 15.2 ±  14.0 mm | F = 0.4 p = 0.03 |

**(a) Right stimulation site connectivity**


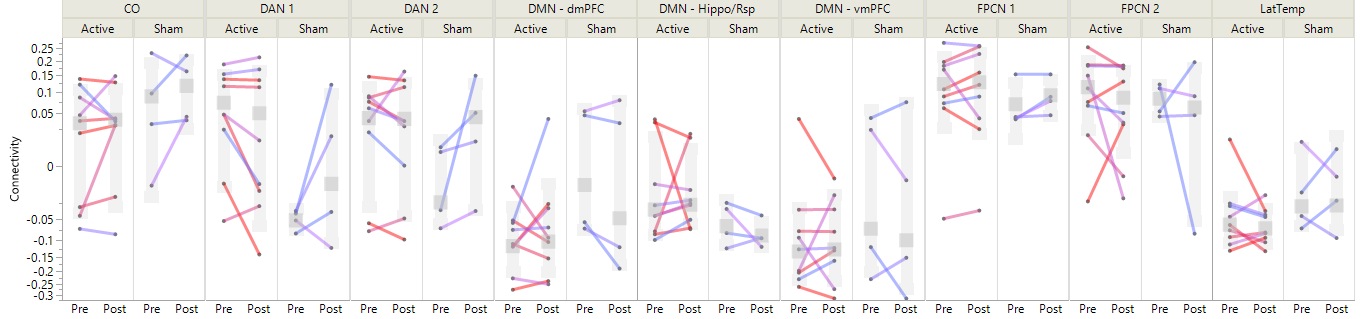

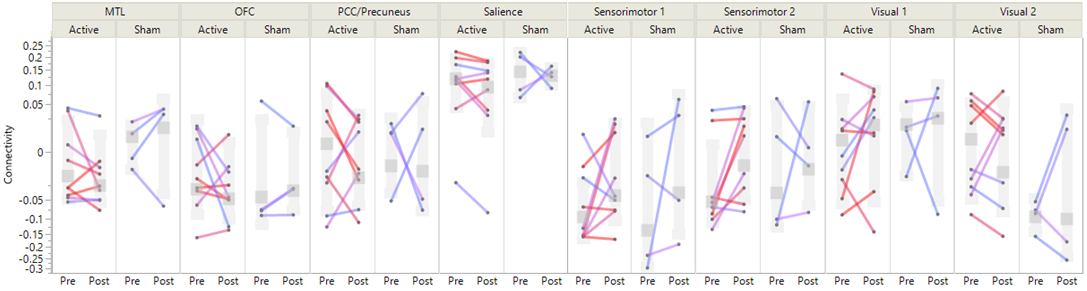

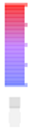


70%

50%

30%

**Percentage
improvement
in MADRS**

Mean
SD

**(b) Left stimulation site connectivity**


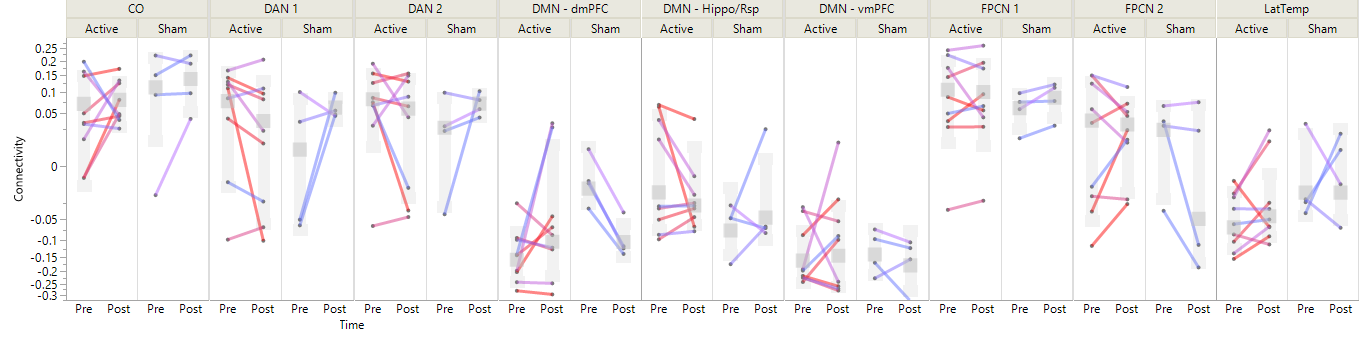

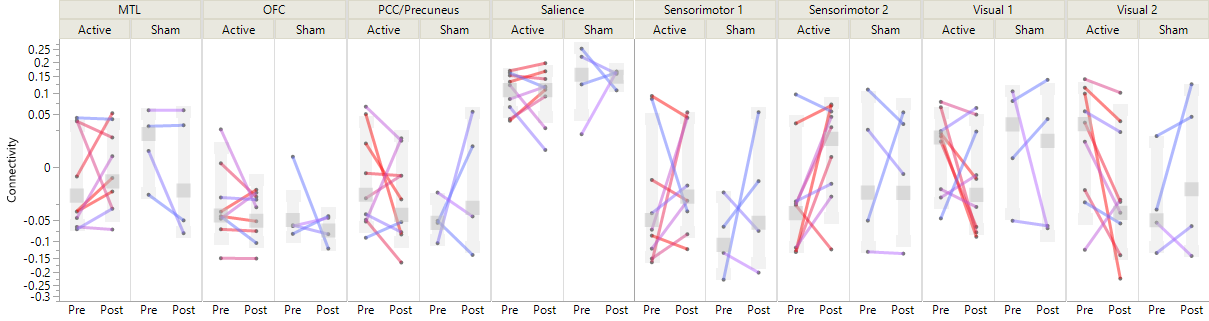

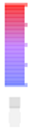


70%

50%

30%

**Percentage
improvement
in MADRS**

Mean
SD

**Figure S2: Stimulation site connectivity and antidepressant response.** (a) Right-sided and (b) left-sided stimulation site connectivity with 17 Yeo network maps before and after treatment in both treatment groups. Colors represent overall antidepressant efficacy (change in MADRS).


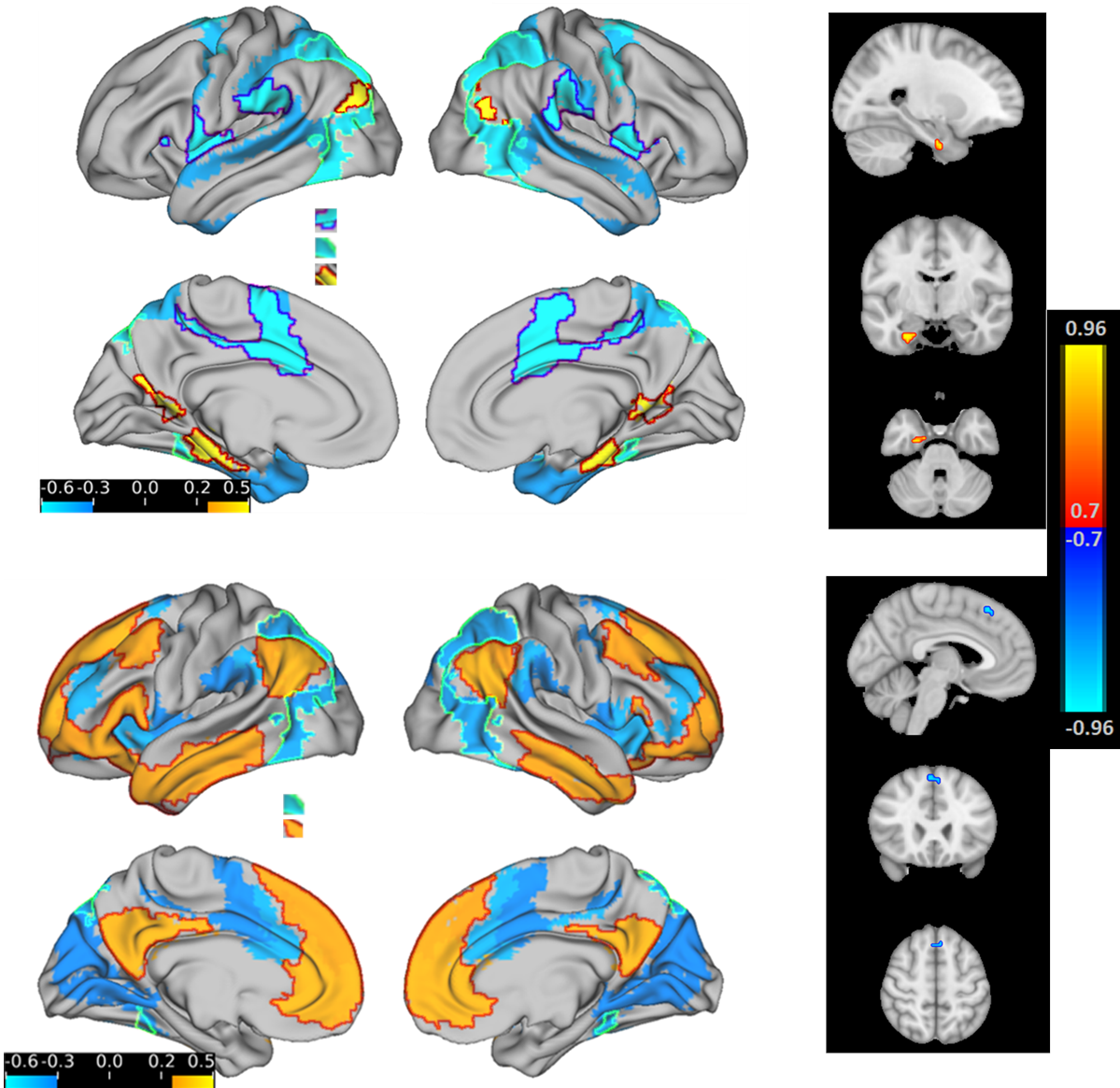


**(a) (b)**

**(c) (d)**

**Cingulo-opercular**

**Parieto-occipital DAN**

**Parahippocampal/retrosplenial DMN**

**Parieto-occipital DAN**

**Prefrontal/parietal DMN**

**Right stimulation site connectivity change (*rho*)**

**Left stimulation site connectivity change (*rho*)**

**L R**

**L R**

**Figure S3: Change in stimulation site connectivity with Yeo network maps and with whole brain.** Red arrows depict left and right stimulation sites. **(a)** Change in right stimulation site connectivity with 3 of the 17 Yeo networks. Outlines depict regions for which the absolute effect size (Spearman rho) was greater than 0.5. **(b)** Change in right stimulation site connectivity with whole brain after cluster-wise multiple comparisons correction (detection threshold p<0.001, cluster extent threshold 351 mm^3^). **(c)** Change in left stimulation site connectivity with 2 of the 17 Yeo networks. The two highlighted networks showed the greatest change. **(d)** Change in left stimulation site connectivity with whole brain after cluster-wise multiple comparisons correction.

# Supplementary References

1. Siddiqi, S.H. *rTMS for major depression associated with TBI*. 2016; Available from: osf.io/vjddq.

2. Siddiqi, S.H., et al., *Repetitive Transcranial Magnetic Stimulation with Resting-State Network Targeting for Treatment-Resistant Depression in Traumatic Brain Injury: A Randomized, Controlled, Double-Blinded Pilot Study.* J Neurotrauma, 2019. **36**(8): p. 1361-1374.

3. Van Essen, D.C., et al., *The Human Connectome Project: a data acquisition perspective.* Neuroimage, 2012. **62**(4): p. 2222-31.

4. Gordon, E.M., et al., *Precision Functional Mapping of Individual Human Brains.* Neuron, 2017. **95**(4): p. 791-807 e7.

5. Laumann, T.O., et al., *Functional System and Areal Organization of a Highly Sampled Individual Human Brain.* Neuron, 2015. **87**(3): p. 657-70.

6. Reuter, M., et al., *Within-subject template estimation for unbiased longitudinal image analysis.* Neuroimage, 2012. **61**(4): p. 1402-1418.

7. Power, J.D., et al., *Methods to detect, characterize, and remove motion artifact in resting state fMRI.* Neuroimage, 2014. **84**: p. 320-41.

8. Siddiqi, S.H., et al., *Individualized Connectome-Targeted Transcranial Magnetic Stimulation for Neuropsychiatric Sequelae of Repetitive Traumatic Brain Injury in a Retired NFL Player.* J Neuropsychiatry Clin Neurosci, 2019. **31**(3): p. 254-263.

9. Hacker, C.D., et al., *Resting state network estimation in individual subjects.* Neuroimage, 2013. **82**: p. 616-633.

10. Marcus, D.S., et al., *Informatics and data mining tools and strategies for the human connectome project.* Front Neuroinform, 2011. **5**: p. 4.

11. Weigand, A., et al., *Prospective Validation That Subgenual Connectivity Predicts Antidepressant Efficacy of Transcranial Magnetic Stimulation Sites.* Biol Psychiatry, 2018. **84**(1): p. 28-37.

12. Fox, M.D., et al., *Efficacy of transcranial magnetic stimulation targets for depression is related to intrinsic functional connectivity with the subgenual cingulate.* Biol Psychiatry, 2012. **72**(7): p. 595-603.

13. Jenkinson, M., et al., *FSL.* Neuroimage, 2012. **62**(2): p. 782-790.

14. Bullmore, E.T., et al., *Global, voxel, and cluster tests, by theory and permutation, for a difference between two groups of structural MR images of the brain.* IEEE transactions on medical imaging, 1999. **18**(1): p. 32-42.

15. Mir-Moghtadaei, A., et al., *Concordance Between BeamF3 and MRI-neuronavigated Target Sites for Repetitive Transcranial Magnetic Stimulation of the Left Dorsolateral Prefrontal Cortex.* Brain Stimul, 2015. **8**(5): p. 965-973.

16. Fox, M.D., H. Liu, and A. Pascual-Leone, *Identification of reproducible individualized targets for treatment of depression with TMS based on intrinsic connectivity.* Neuroimage, 2013. **66**: p. 151-60.

17. Jenkinson, M., et al., *FSL.* Neuroimage, 2011. **62**(2): p. 782-790.

18. Opitz, A., et al., *An integrated framework for targeting functional networks via transcranial magnetic stimulation.* Neuroimage, 2016. **127**: p. 86-96.

19. Fox, M.D., et al., *Resting-state networks link invasive and noninvasive brain stimulation across diverse psychiatric and neurological diseases.* Proc Natl Acad Sci U S A, 2014. **111**(41): p. E4367-75.

20. Yeo, B.T., et al., *The organization of the human cerebral cortex estimated by intrinsic functional connectivity.* J Neurophysiol, 2011. **106**(3): p. 1125-1165.

21. Gordon, E.M., et al., *Generation and Evaluation of a Cortical Area Parcellation from Resting-State Correlations.* Cerebral cortex, 2016. **26**(1): p. 288-303.

22. Slotnick, S.D., *Cluster success: fMRI inferences for spatial extent have acceptable false-positive rates.* Cogn Neurosci, 2017. **8**(3): p. 150-155.

23. Eklund, A., T.E. Nichols, and H. Knutsson, *Cluster failure: Why fMRI inferences for spatial extent have inflated false-positive rates.* Proc Natl Acad Sci U S A, 2016. **113**(28): p. 7900-5.
